# Prediction model of free flaps in microkeratome-assisted LASIK

**DOI:** 10.1371/journal.pone.0255525

**Published:** 2021-09-01

**Authors:** Toam Katz, Vasyl Druckiv, Sebastian Siebelmann, Andreas Frings, Christos Skevas

**Affiliations:** 1 Care Vision Academy, Germany; 2 Department of Ophthalmology, University Hospital Hamburg Eppendorf, Hamburg, Germany; 3 Clinica Baviera, Valencia, Spain; 4 Department of Ophthalmology, University Hospital of Cologne, Valencia, Germany; 5 Department of Ophthalmology, University Hospital Düsseldorf, Duesseldorf, Germany; 6 Augenarztpraxis Dr. Frings, Nürnberg, Germany; University of Toronto, CANADA

## Abstract

**Purpose:**

To identify mechanical factors, as well as patients’ biometric and surgeons’ experience factors that correlate with the FF incidence in microkeratome (MK)-assisted LASIK and to construct a predictive model based on these parameters.

**Methods:**

55,700 consecutive LASIK treatments of 28,506 patients between January 2017 and April 2020 done by 50 surgeons in 10 centers, all with Sub Bowman Keratome (SBK) and 90μ head (OUP) were analyzed retrospectively for the incidence of FF and their correlation to mean keratometry, central corneal thickness, MK ring height and stop, as well as surgeons’ experience. A prediction model was built and tested for sensitivity and specificity.

**Results:**

The incidence of FF using the SBK MK was 0.276%. Risk factors were low central corneal thickness, very flat (-1) or very thick (+2) ring height, and higher stop values (p<0.001). Mean keratometry and low surgeon experience were not correlated to FF incidence. A prediction model with a cut-off FF risk of 0.274%, a 76% specificity, and a 73% sensitivity was applied.

**Conclusions:**

Free flaps are rarely seen in modern MK LASIK. However, the incidence of this complication using the SBK MK increases using higher stop values, very thick and very thin MK rings, and in eyes with thin corneas.

## Introduction

Laser in Situ Keratomileusis (LASIK) needs a precise and stable flap. This flap is created either by a microkeratome (MK) or by a femtosecond laser (FSL) and includes a tissue-bridge (Hinge) either nasal or superior to the ablation zone to secure a correct repositioning and stabilization of the flap.

The most common among the generally rare intraoperative flap complications is an unintended cut through the intended hinge, which produces a mobile free cap or free flap (FF). Such a complication makes the ablation more difficult and may lead to decentration and rotation of the repositioned flap, flap folds and epithelial ingrowth, irregular astigmatism, as well as a complete loss of the flap, either intra- or post-operatively.

Expanding the range of keratectomy to steeper and flatter corneas while aiming at a large ablation zone and still producing a stable hinge is the goal of modern MK. However, even the best instrumentations and nomograms cannot guarantee a perfect flap. Human factors such as narrow palpebral aperture or a stressed patient might cause a ring vacuum loss and a variety of flap complications, including an FF [1].

**Fig 1:**
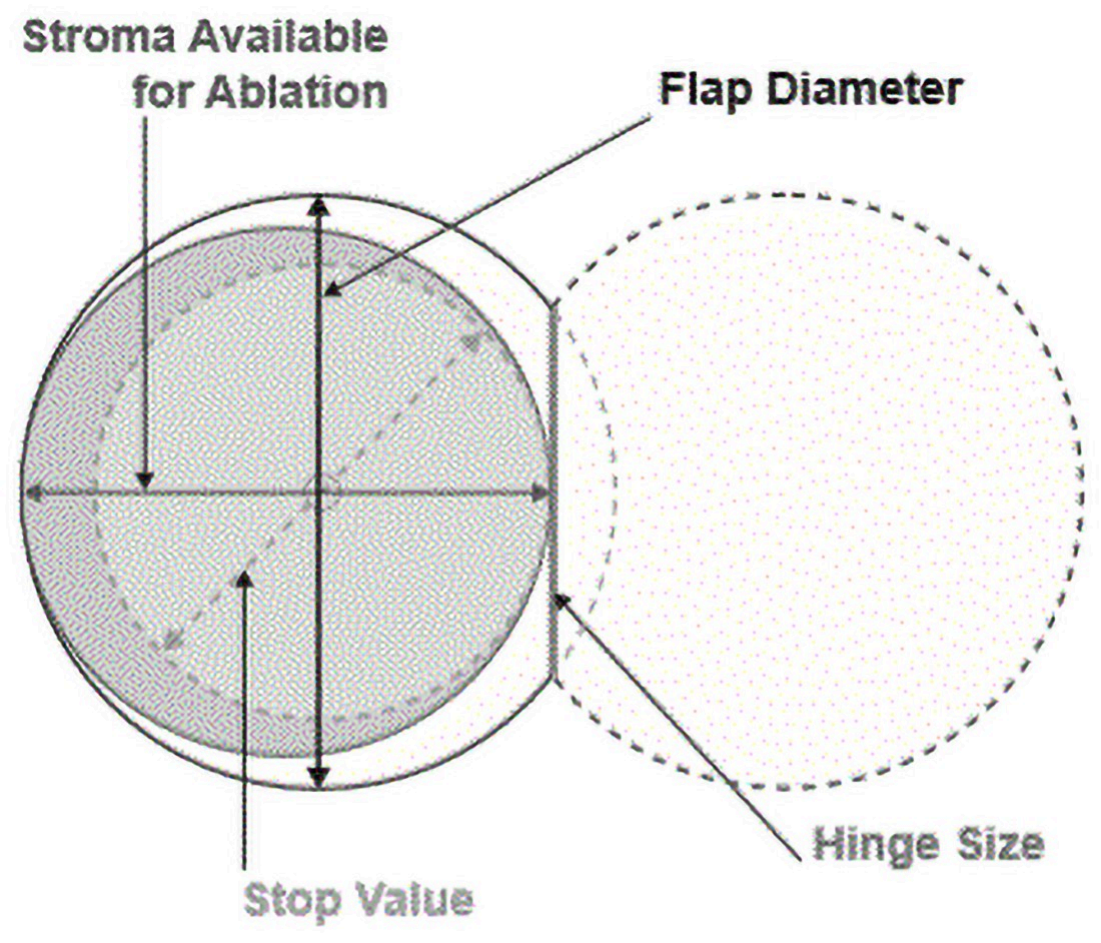
Ring and stop in microkeratome flap.

**Fig 2:**
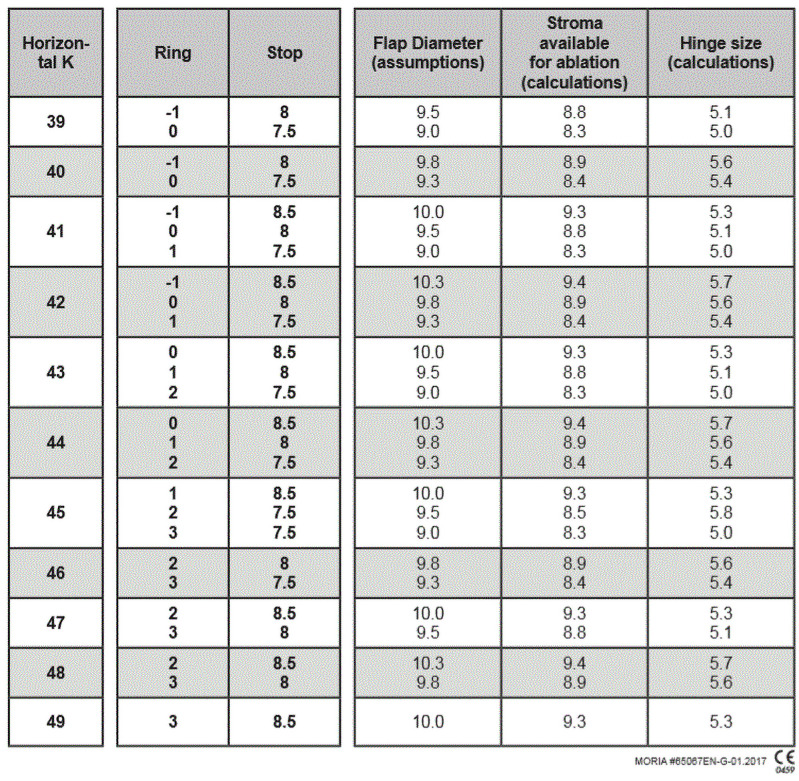
Expected flap size for rings and stops per microkeratome nomogram.

The user manual of the MK in this study recommends using a specific thickness (height) of the vacuum ring according to the horizontal keratometry of the cornea. An average cornea has a horizontal keratometry of 43.00 D and should be cut with a ring height named (0), while flatter corneas should be cut with a flatter ring marked (-1) and steeper corneas using a thicker ring marked (+1) or (+2). For each ring thickness and keratometry the manufacturer recommends a stop value between 7.5 and 8.5 mm, which predicts the horizontal diameter of the flap. For a given keratometry, a lower ring value will produce a larger flap diameter and a smaller hinge. A too high stop value will cut through the hinge unintendedly and create a free flap. A too low stop value will avoid an FF but may cause a too short horizontal flap diameter with a hinge within the intended ablation zone (Fig 1). For each keratometry there is an overlap of two to three different proposed ring and stop combinations which differ in the flap and hinge size. Very large (> 12 mm) or very small (< 11 mm) horizontal corneal diameter (white-to-white) should be cut with a lower or higher ring size (Fig 2). All recommended settings should produce a sufficient ablation zone with a stable hinge. The surgeon uses a vacuum pump (Evo3, Moria, Antony, France) to fixate the MK ring on the conjunctiva. Miscalculation of the ring and stop combination even under a correct vacuum may also cause a too short flap or the opposite–a hinge-less flap.

The purpose of this retrospective large-scale cross-sectional study was to identify parameters that can predict the incidence of an FF in microkeratome-assisted LASIK.

## Material and methods

This retrospective study analyzes the incidence of FF in 55,155 eyes of 28,247 patients who have had LASIK for myopia, hyperopia and astigmatism as a primary procedure or as a touch-up after previous lens implantation in 10 refractive surgery centers by 50 different surgeons between January 2017 and April 2020.

The guidelines of the Declaration of Helsinki have been respected. After consulting the ethics committees, a vote was not required because it was a retrospective data collection that was anonymized at the source.

All eyes were analyzed by corneal topography using a Scheimpflug tomographer (Pentacam, Oculus, Germany) and were treated using an automated MK (Sub Bowman Keratectomy, SBK), the Evolution 3 vacuum system with single use vacuum tube, and a single-use MK head (One Use Plus, OUP 90μm), all produced as a system by the same manufacturer (Moria, Antony, France) and used as recommended by the manufacturer. All treatments were planned on an electronic form including pre-set combinations of ring and stop. The surgeon had to choose one preset combination for the planning. This choice is imported into the treatment form and checked manually and verbally between the surgeon and the laser assistant. The actual used combination as well as all treatment data was saved in the data bank and extracted for this study. The combination of ring -1 and stop 8.5 is extreme but is recommended by the manufacturer for the largest flaps for horizontal keratometry of 41 D and 42 D. This combination appears in “Nomogram for one use plus with metal ring.”

The pretesting and usage of the LASIK instruments followed a surgical protocol. The central corneal thickness (CCT) was measured intraoperatively with an ultrasound pachymeter (Pachette3, DGH technology, PA, USA or SP-100, Tomey, Japan) or a non-contact pachymeter built into the laser platform (Wavelight EX500, Alcon, USA).

Any intraoperative complication including a free flap as well as the pre-operative and intra-operative parameters was documented in the electronic medical file and retrieved retrospectively.

The following parameters of 55,155 consecutive treatments including the FF eyes and the uncomplicated MK-LASIKs were compared: ring size (-1,0, +1, +2), stop value (7.5, 8.0, 8,5), mean keratometry (38–48 D), minimal corneal thickness (466 to 734 μm) and the LASIK experience (N) of the 50 surgeons (mean 4,405 LASIKs ± 3,793) prior to the occurrence of FF case. The demographics and overall incidence of FF are shown in Table 1.

**Table 1:** Demographics of free flap eyes.

Patients	28.247
Surgeons	50
Eyes	
Right	27,655 (50%)
Left	27,500 (50%)
Sex	
Female	16,307 (58%)
Male	11,940 (42%)
Age (years)	17 to 79; 33 (±6)
Manifest Refraction	
Hyperopia	5,118 (9%)
Myopia	50,012 (91%)
Keratometry (D)	38 to 48; 44 (±1)
Central corneal thickness (CCT) (μm)	466 to 734; 559 (±20)
Nr of LASIKs	1 to 18978; 4393 (±3794)
Free Flap	
No	55,003 (99.724%)
Yes	152 (0.276%)

## Statistical analysis

Data were extracted from the SQL database and processed and analyzed using R Core Team (2019).^1^ To estimate the differences in FF incidence between different types of rings and stops, the logistic regression model was fitted. When analyzing the effect of keratometry on the corneal thickness with different stops, another logistic regression was performed.

The relationship between ring, stop and incidence of FF was further analyzed by a model using logistic regression in relation to ring size (-1) and a stop of 7.5 mm.

Note that log-odds [p/(1−p)] can be transformed to odds ratios (OR) by exponentiating the coeficients (β-s). Odds ratios and p-values from these models are reported. The formal definition of the model is the following:

$$\text{Log}\;\left[\text{p}/\left(1-\text{p}\right)\right]=\beta_{0}+\beta_{\text{ring}0}+\beta_{\text{ring}+1}+\beta_{\text{ring}+2}+\beta_{\text{stop}8}+\beta_{\text{stop}8.5}$$

The incidence of FF was compared to the amount of LASIKs (N) performed by the surgeon before the FF case using a non-linear regression.

To create and test a predictive model, the following variables were used: ring, stop, mean keratometry, CCT. The incidences of FF per ring size were compared to flatter ring (-1). The incidences of FF per stop values were compared to a shorter stop of 7.5 mm. Mean keratometry and CCT are calculated based on a training subgroup and used for a regression model. We randomly selected two thirds of the sample (36,402 LASIKs) for training the model and saved one third of the sample (18,753 LASIKs) for testing the model for specificity and sensibility. An arbitrary cut-off value of FF incidence that allows for good specificity and sensibility was chosen.

## Results

**Fig 3:**
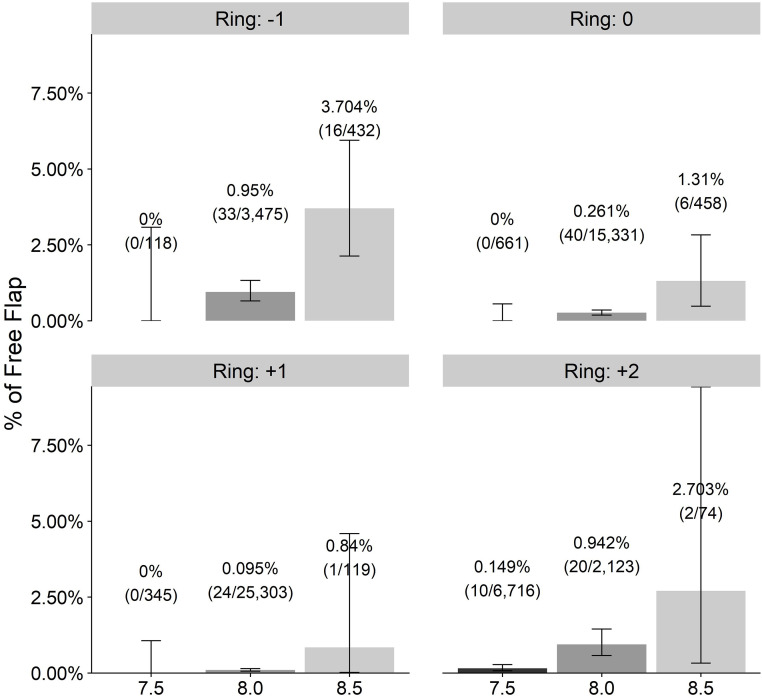
Free flap incidence per rings and stops.

In this study, the overall incidence of FF using the SBK and OUP MK in 40 months was 0.276% (152 FF from 55,155 LASIK eyes). As expected, the incidence of FF by ring and stop was higher for all rings when a high stop value was chosen. The flattest ring (-1) and the thickest (+2) produced more FFs than the (0) and (+1) rings when using 8.0 or 8.5 mm stops. Ring (+2) produced FFs even with a very short stop of 7.5 mm (Fig 3).

The relationship between ring, stop and incidence of FF analyzed by logistic regression is shown in Table 2.

**Table 2:** Odds ratios of rings, stops and free flap incidence.

Contrast	Odds Ratio *	P-Value †
Stop		
(8)–(7.5)	6,59	<0.001
(8.5)–(7.5)	28,02	<0.001
(8.5)–(8)	4,25	<0.001
Ring		
(0)–(-1)	0,28	<0.001
(+1)–(-1)	0,1	<0.001
(+2)–(-1)	1	1
(+1)–(0)	0,36	<0.001
(+2)–(0)	3,52	<0.001
(+2)–(+1)	9,66	<0.001

* P-Values obtained using post hoc Tukey test after logistic regression.

^†^ Odds ratios from differences of marginal effects.

All stops differ significantly in FF incidence from each other. Wald test p<0.001 was applied. P values for all contrasts are <0.001 as well. The contrasts for stops are constructed such that a smaller stop is subtracted from a greater stop. The overall effect of stop is significant. For instance, the odds for FF with stop 8.5 are 4.25 times higher than the odds for free flap with stop 8.0.

For rings, the interpretation is similar. The overall effect of rings was significant, with Wald test p<0.001. All contrasts are significant except the difference between high FF incidence (+2) and (-1) rings. For instance, the FF incidence using a (-1) ring is larger than when a (0) ring was applied. The odds for free flap with a (-1) ring are 1/OR(0) = 1/0.28 = 3.21 times higher than the odds of free flap with a 0 ring. Or, alternatively, the odds in (-1) ring cases are 3.6 times higher than the odds in (0) ring (1/0.28 = 3.6).

The incidence of FF by keratometry and stop using the same ring, in this example ring (+2), shows a much higher FF incidence for higher stop (OR = 5.45). The same method was applied for testing the hypothesis that keratometry is related to free flap incidence to find out that a flatter mean keratometry using either stop did not increase the incidence of FF significantly (p = 0.356). For example, the ring (+2) that may be used in keratometries from 43 to 48 D with the recommended stops and should not have caused an FF did cause significantly more FF in corneas steeper than 45.5 D (Table 3).

**Table 3:** Odds ratios of keratometry and free flap incidence.

Contrast	Odds Ratio *	P-Value †
Km		
[45.5,46,5)–[44.5;45.5)	0,65	0,356
Stop		
(8)–(7.5)	5,45	<0.001

* P-Values obtained using post hoc Tukey test after logistic regression.

^†^ Odds ratios from differences of marginal effects.

The central corneal thickness in the free flap group was 551.17 ± 30.79 (N = 152), while in the group without complications, the mean total thickness was 560.56 ± 31.04 (N = 54,823), t test p<0.001, suggesting a trend in free flap eyes towards thinner CCT. Unfortunately, some eyes had missing values of CCT as documented in the electronic file intraoperatively and were excluded.

This result is confirmed with a logistic regression that gives an odds ratio of 0.99 (95% CI: [0.984;0.995]), p<0.001. For instance, the eyes with CCT of 500μ have 2.833 higher risk of free flap than the eyes with CCT of 600μ.

**Fig 4:**
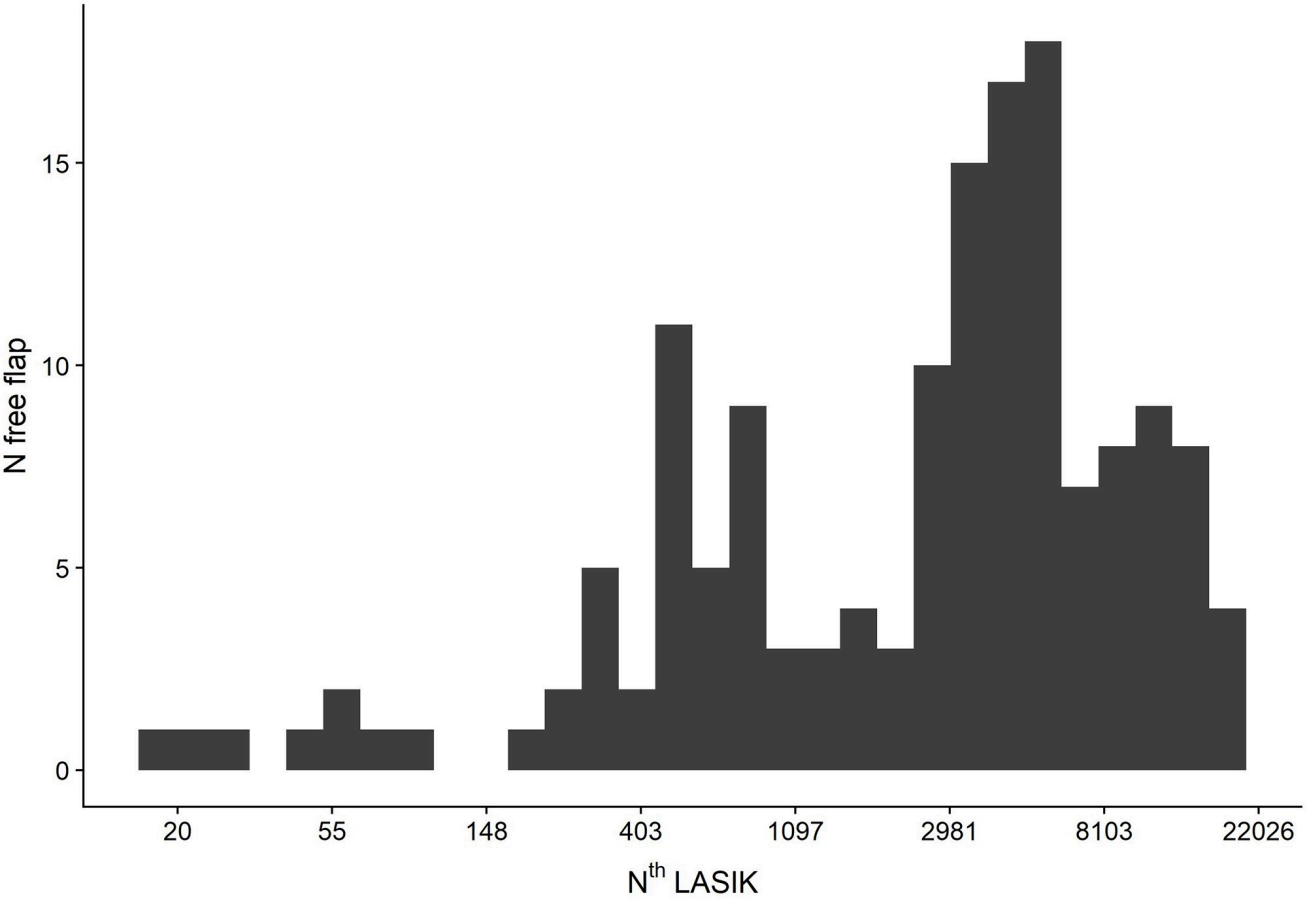
Surgeons’ experience and free flap incidence.

Most of the 50 LASIK surgeons gained their surgical experience before the beginning of the study. Prior to their FF case, the surgeons had performed between 1 and 18,978 LASIKs (Mean 4,393 +-3,794 LASIKs) (Table 1). Fig 4 shows how many free flaps occurred in specific bins of N^ths^ treatments. In some cases it occurred around the 20th LASIK, but in most of the cases, it happened to surgeons with a high level of experience. Specifically, most free flaps occurred between Median (Q25/Q75): 3,482 (844, 5,580) LASIK treatments. Of course the surgeons with high n^th^ LASIK do many more LASIKs than surgeons whose n^th^ LASIKs are <1,000, but this figure demonstrates that free flaps can occur at any level of experience.

**Fig 5:**
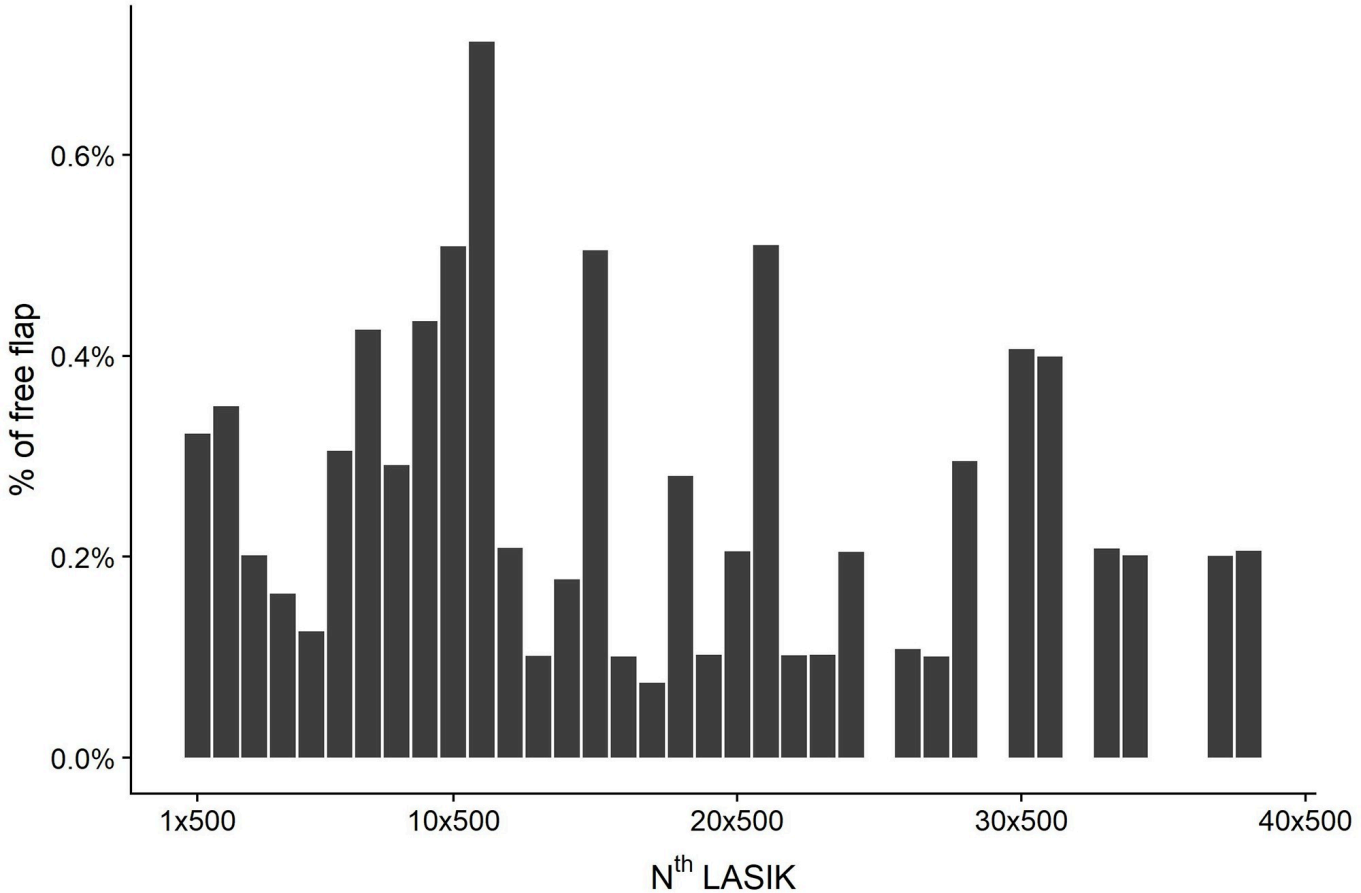
LASIK bins per surgeon and free flap incidence.

In order to investigate the FF incidence related to the total (N) LASIKs per surgeon, the experience was divided into bins of 500 LASIKs each (Fig 5). For instance, the first bar shows the precentage of free flaps between 1 and 500 LASIKs, the second between 501 and 1,000 LASIKs, the third between 1,001 and 1,500 LASIKs, etc. As of January 2017, 28 surgeons started with treatments numbering less than 500 and 22 surgeons had already done more then 500 LASIKs.

No trend was seen for more FF in the outermost left columns. Neither logistic regression model showed any significant correlation between n^th^ LASIK treatment and occurence of FF. It seems that the number of treatments doesn’t relate to free flap incidence. Furthermore, the logistic regression with free flap as a dependent variable and logged n^th^ LASIK treatment as an independent variable didn’t detect any significant correlation, p = 0.126. Surprisingly, none of several models of regression analysis found any correlation between the surgical experience of the surgeon and the incidence of the FF.

### Predictive incidence model

To create and test a predictive model, the following variables were used: ring, stop, mean keratometry and CCT. The incidences of FF per ring size compared to flatter ring (-1), stop compared to shortest stop of 7.5 mm, mean keratometry, and central corneal thickness are calculated based on a training subgroup (Table 4) and were used for a regression model as follows:

**Table 4:** Odds ratios of central corneal thickness and free flap incidence.

	Dependent variable:
	Incidence of free flap
KM (D)	-0.674***
	-0,125
Ring (0)	-0.515*
	-0,303
Ring (+1)	-0,678
	-0,458
Ring (+2)	3.064***
	-0,621
Stop (8)	2.415***
	-0,443
Stop (8.5)	4.142***
	-0,556
Log (N tts)	-0.014***
	-0,003
Constant	28.543***
	-5,645
Observations	36.277
Log Likelihood	-619,21
Akaike Inf. Crit.	1.254,42

*p<0.1;

**p<0.05;

***p<0.01.

Two thirds of the sample was selected for training the model, and one third of the sample was saved for testing the model for specificity and sensibility. The multi variance logistic regression model on the training data set produced the following logit equation:

$$\begin{array}{cc} Logit(Free\;Flap)\;=\; & 28.543-0.674\times K(D)\\ & -0.515\times Ring(0)-0.678\times Ring(+1)+3.064\times Ring(+2)\\ & +2.415\times Stop(8)+4.142\times Stop(8.5)\\ & -0.014\times MCT\;(\mu ) \end{array}$$

Logits can be transformed to probabilities as follows:

$$\begin{array}{rr} p & \;=\;\frac{e^{Logit(Free\;Flap)}}{1+e^{Logit(Free\;Flap)}} \end{array}$$

In this model, a value “1” is inserted for a stop and ring in use, and a value “0” is inserted for not used rings and stops. The keratometry will be inserted in diopters (D) and the corneal thickness in micrometers (μ).

For a realistic example of low FF risk, this model predicts that a mean keratometry of 43 D using ring +1 and stop 8 on a 600 micrometer thick cornea should be calculated as:

$${\text{Logit}}_{\left(\text{FF}\right)}=28,543–0.674\;\text{x}\;43-0.678\;\text{x}\left(1\right)+2.415\;\text{x}\left(1\right)–0.014\;\text{x}\;600=-7.097$$

$$\text{P}={\text{e}}^{-7.097}/\left(1+{\text{e}}^{-7.097}\right)=0.000827=0.087\%\;\text{or}\;1\;\text{to}\;1149\;\text{LASIKs}\;\text{predicted}\;\text{FF}\;\text{risk}.$$

While a realistic high risk combination using a ring of (+2) with stop 8.5 on a steep 47 D and 520 micrometer thin cornea will predict:

$${\text{Logit}}_{\left(\text{FF}\right)}=28,543–0.674\;\text{x}47+3.064\;\text{x}\left(1\right)+4.142\;\text{x}\left(1\right)–0.014\;\text{x}\;520=-3.209$$

$$\text{P}={\text{e}}^{-3.209}/\left(1+{\text{e}}^{-3.209}\right)=0.0421=4.02\;\%\;\text{or}\;1\;\text{to}\;25\;\text{LASIKs}\;\text{predicted}\;\text{FF}\;\text{risk}.$$

**Fig 6:**
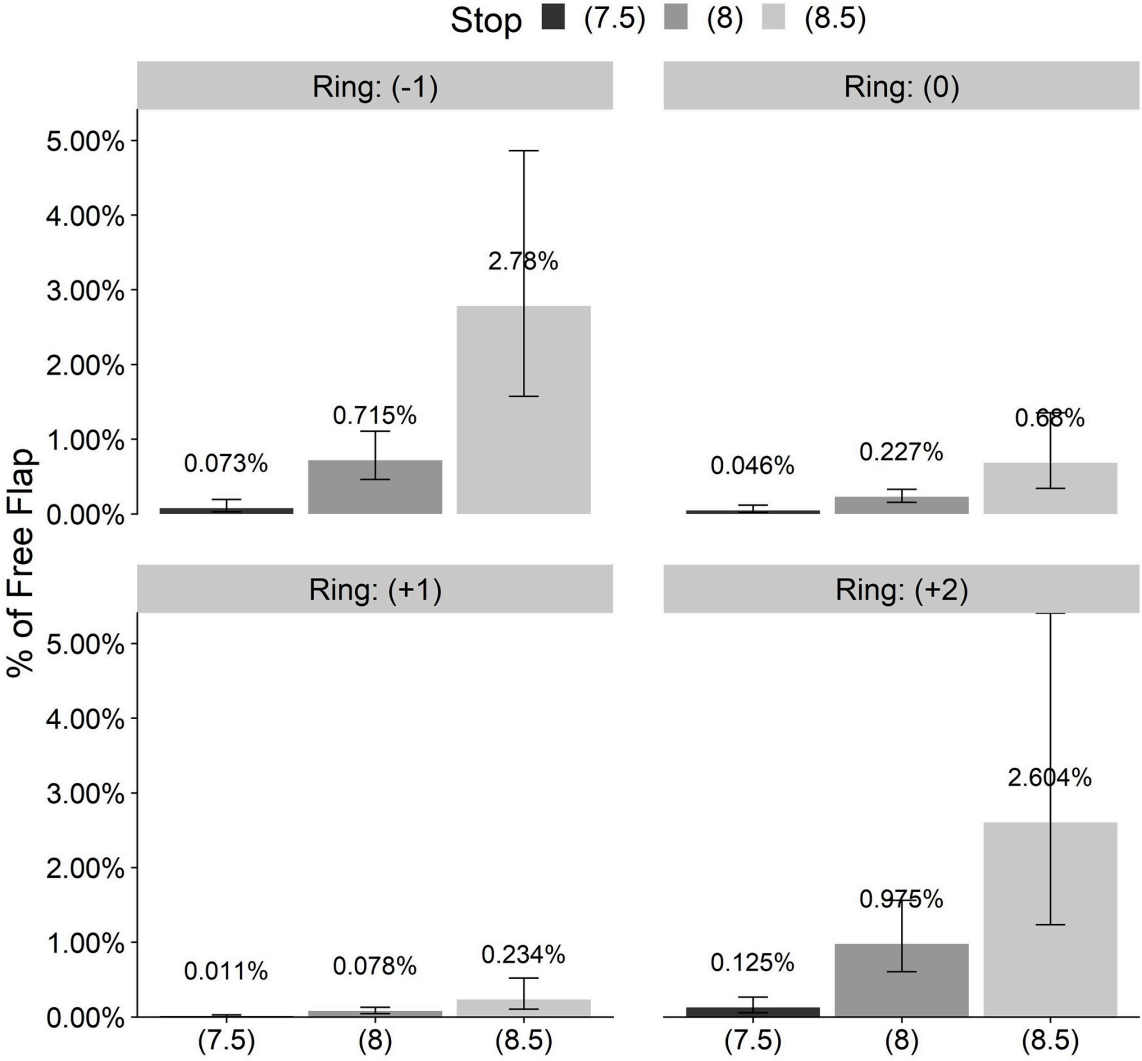
Predicted incidence of free flap by ring and stop.

This prediction model based on two thirds of the eyes can be used to calculate the FF risk depending on two dependent and two fixed total mean variables:

Fig 6 shows the model-predicted incidence of FF for various ring and stop values for the mean values of keratometry (mean K = 44 D) and mean central corneal thickness (M = 559μ).

**Fig 7:**
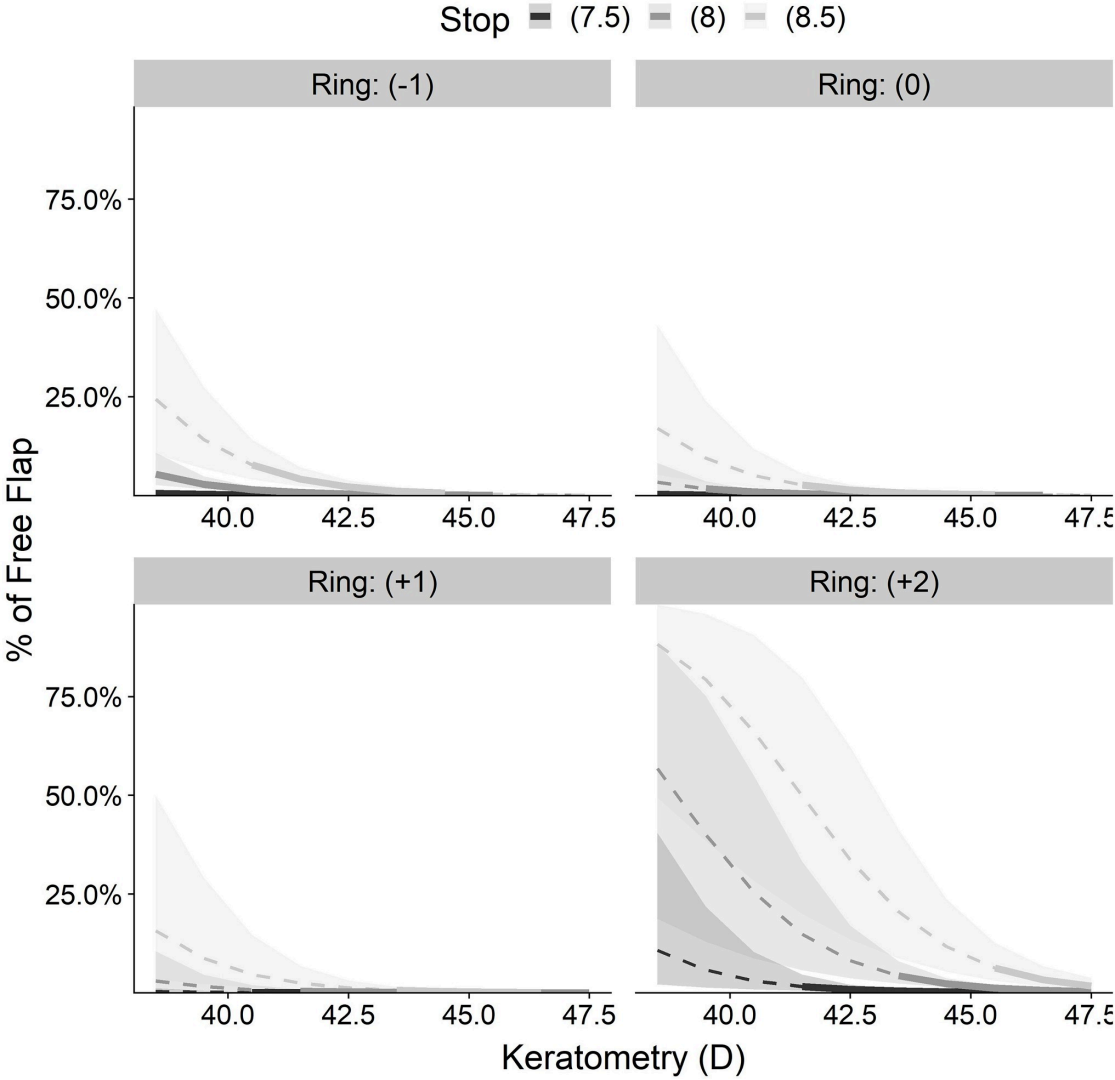
Predicted incidence of free flap by keratometry.

Fig 7 shows the predicted probability for FF by keratometry for each ring and stop combination with total mean corneal thickness (559 μ). The surgeons’ experience is set to an overall mean of N = 4393 LASIKs. The statistical extrapolation also demonstrated the expected effect of the extremly contraindicated combinations that should never be used. The manufacturer’s recommended keratometry range for each ring (Table 5) is marked in Fig 7 as thick lines.

**Table 5:** Multivariance logistic regression for keratometries, rings, stops and CCT of the training data set.

Ring	Stop	Min K	Max K
(-1)	-7,5	38,5	45,55
(-1)	-8	38,5	45,5
(-1)	-8,5	40,25	44,5
0	-7,5	38,5	45,25
0	-8	39,5	46,5
0	-8,5	41,25	45,9
(+1)	-7,5	40,25	45,75
(+1)	-8	40,75	47,5
(+1)	-8,5	43,25	46,5
(+2)	-7,5	41,5	48,25
(+2)	-8	42,8	48
(+2)	-8,5	45,49	48,4

**Fig 8:**
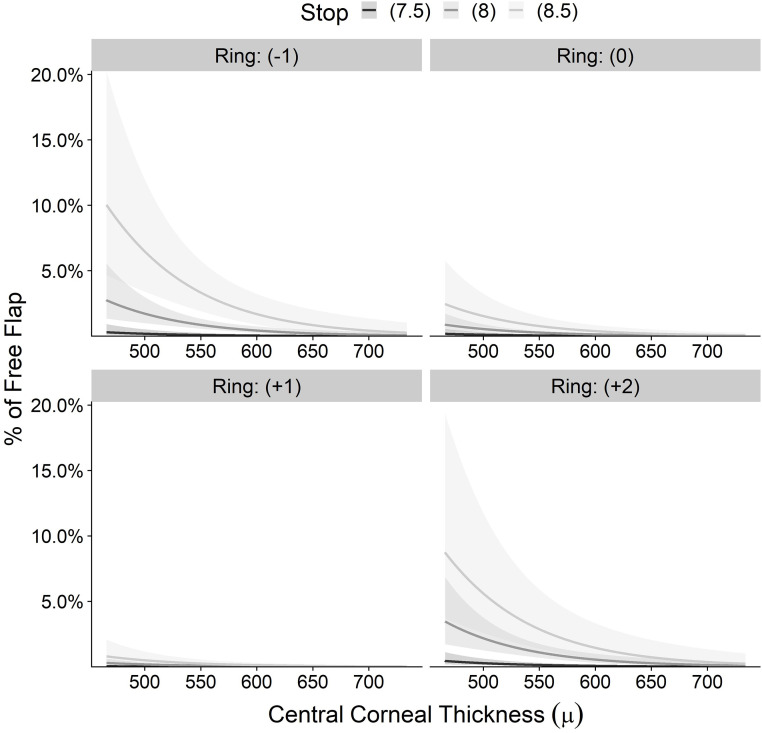
Predicted incidence of free flap by central corneal thickness.

Fig 8 shows the estimated probability for FF by minimal corneal thickness for each ring and stop combination. The keratometry is set to an overall mean of 44 D. The surgeons’ experience is set to an overall mean of N = 4,393 LASIKs.

This model can be used to determine what CCT should exist in order to keep the incidence of FF below 0.274% for a combination of ring (+2), stop 8.5 and mean keratometry of 49.9 D, which is the mean keratometry for this ring and stop combination.

Solving for CCT gives:

$$\begin{array}{r} \frac{log(\frac{0.003}{1-0.003})-A}{-0.014}\;=\;MCT\;(\mu ) \end{array}$$

Putting in A the combination of ring +2, stop 8.5 and keratometry 46.9 gives

$$\begin{array}{r} MCT\;(\mu )\;=\;\frac{log(\frac{0.003}{1-0.003})-(28.543-0.674\times 46.9+3.064+4.142)}{-0.014}\;=\;710 \end{array}$$

So, given K = 46.9, ring(+2) and stop 8.5 per the model, the CCT equal or superior to 710 μ is estimated to give the probability of free flap below 0.3%. Such an optimistically high CCT is very rare in clinical practice.

#### Verification of the regression model

**Fig 9:**
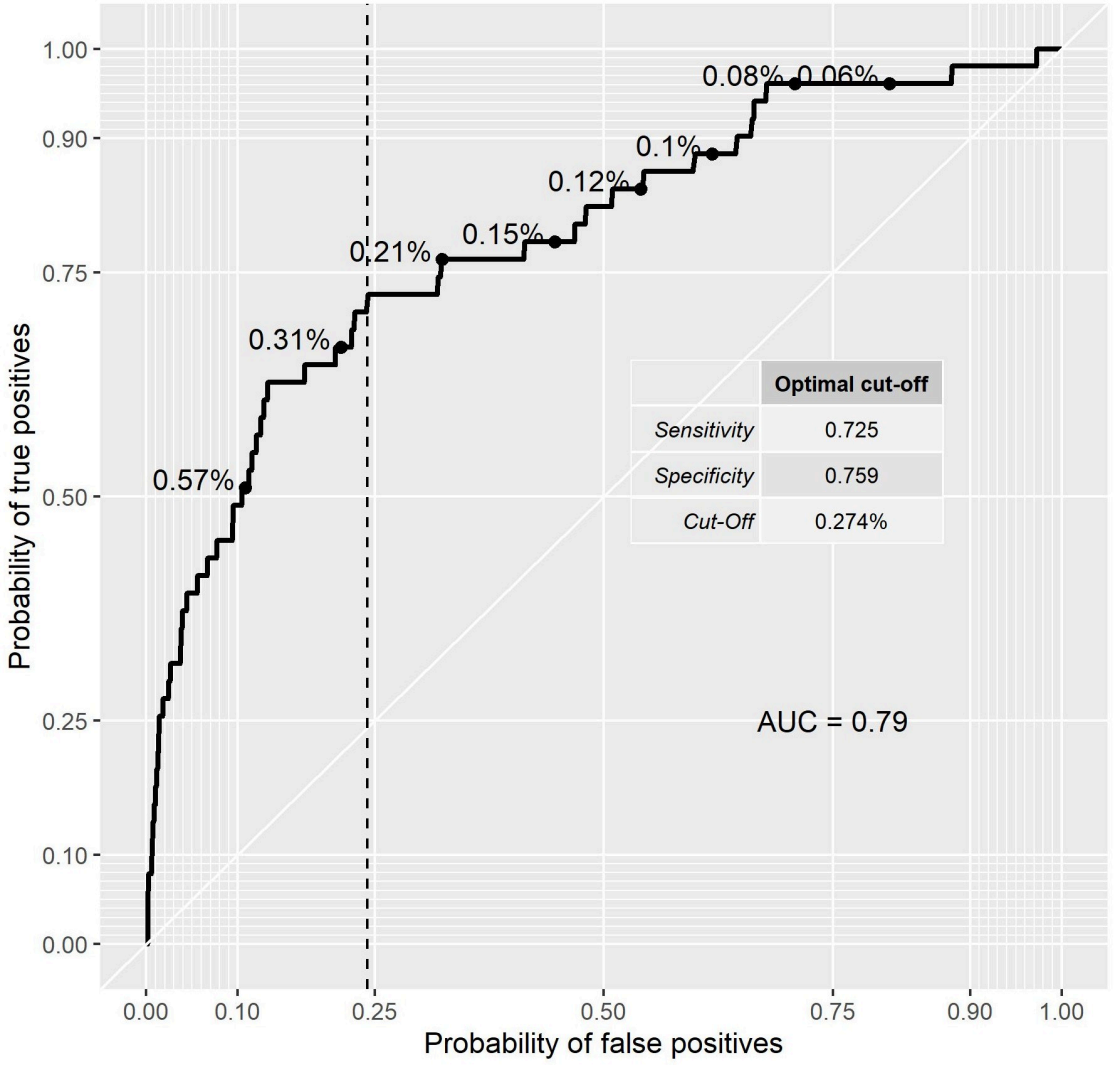
Sensitivity and specificity of prediction model on test data set.

This model was used for the prediction of free flap cases on a test data set of the remaining one third of the eyes. Since the data is strongly unbalanced, i.e. there are many more complication-free eyes than FFs (18,702 [99.73%] normal vs. 51 [0.27%]), the resulting probabilities are very low. Nevertheless, ROC analysis (Fig 9) of the resulting probabilities can be used to compute specificities and sensitivities for every cut-off point. This results in the following classifications for the test data set.

The optimal cut-off is just a value that minimizes the difference between specificity and sensitivity, making two types of errors equally important. This cut-off gives a rule (one of many possible): if the expected probability for an FF is equal or above 0.274%, a new case is classified as free flap, and if the expected probability is lower than 0.274% then the new case is classified as normal flap. This theoretic cut-off FF incidence of 0.274% allows for high and similar values of sensitivity (72.5%) and specificity (75.9%).

With the cut-off of 0.274%, the predictive model was able to correctly classify 37 of 51 cases as free flaps (sensitivity 72.5%). On the other hand, the model incorrectly classified 4,488 of normal eyes as free flaps (1-Specificity = 1–0.759 = 0.241 = 24.1%). This false-positive probability of 24.1% is shown as a vertical line in Fig 9. Given the low incidence of free flaps, the real probability for the patient to have free flap when the model predicted it is still very low (Positive Predictive Value (PPV) = 0.8%). This is low because of a large number of regular flap eyes and moderate specificity.

## Discussion

The results of this large-scale study show that thinner corneas and extremely thin or thick ring heights (-1 and +2), combined with higher stop values, correlate to higher FF risk. The manufacturer’s nomogram indicates several ring size options per keratometry. Following this nomogram, the mean corneal keratometry did not contribute per se to the FF risk. Surprisingly, the experience of the surgeons, varying greatly among 50 surgeons, also did not influence the FF incidence.

The study found an FF incidence of 0.276%. The flap-related complications are generally very rare and only few studies discuss the factors causing these complications. No study was found that focuses on FF and its causes. Most studies from the last five years compare different models of MK to femtosecond laser assisted flaps and include only small cohorts of retrospective uncomplicated procedures.

Previous literature suggest that the FF incidence is similar between MK and FSL assisted LASIK. Moshirfar et al. compared intraopertive complications in 896 MK eyes (Hansatom, 160 μm Flap) and 902 FSL (Intralase FS60) eyes and found a similar FF incidence of 0.4% [2,3].

Reports older than 10 years of MK associated complications are based on older models of MKs and small series of cases. Due to the rarety of this complication, a very large series of LASIKs is essential to draw an accurate risk. A search in Pub Med did not recover any report on the incidence of FF in large (>1000) LASIK series published in the last 10 years. In a report from 2016 [4] of 273 LASIK flaps done by 32 young residents using an automated pivoting MK (M2, Moria, France), five out of 273 (1.83%) keratectomies ended with an FF. In another large cohort [5] of 2,883 LASIK eyes using the same SBK MK as the present study, 80 eyes had flat corneas below 40 D and (-1) ring was used, either with 7.5 mm stop (14%) or with 8.0 mm stop (86%); none of the 80 eyes had an FF. In another series [5] of 806 LASIK eyes using the M2 MK, only one eye (0.124%) had an FF.

In older literature using older MKs, the FF rate varies. Those models which are no longer used created flaps that are thinner in the center, and in order to avoid serious complications like a buttonhole the flaps were designed to be very thick, thus increasing the ectasia risk. Carrilo et al.^6^ reported his experience in 2005 using an automated Nidek MK-2000 on 23 out of 26,600 (0.086%) LASIKs in five centers. This MK uses ring sizes of 8.5mm and 9.5 mm, which determines the flap diameter. FF incidence was about a third of the present study’s FF incidence. However, the Nidek MK-2000 created less homogeneous and much thicker flaps with 130μ or 160μ heads with changing thickness, which produced a high rate of serious flap complications like 0.049% incomplete flap, 0.041% buttonhole and 0.019% irregular flaps. Using an older MK model like the Automated Corneal Shaper, a very high 6.7% (6/90) incidence of flap complication including 1/90 FF (1.1%) was reported. The same surgeon performed LASIKs with a Hansatome MK with 2/598 (0.3%) incomplete or crescent flap comlications and no FF [6]. MK design as well as the learning curve could influence this difference in the incidence of complications. Pallikaris et al. reported an extremely high flap complication incidence of 14% (48/334 myopic eyes) using the Mediate Mel 60 microkeratome [7]. Four of the 334 LASIKs (1.2%) resulted in a low risk free flap but all of the other 44 flap complications were worse. In 11 complicated keratectomies, the procedure had to be aborted. The authors found a correlation between thin flaps and high keratometry and high astigmatism but did not find any statistically significant relationship between corneal anatomic factors and keratome-related complications in this series. Most complications were related to intraoperative suction loss. Due to improvement of the MK suction systems, according to the authors, the rate of flap complications decreased from 14% to 1.25% (3/239 eyes).

Considering the mechnism of the MK, it is clear that using the rings and stops other than as recommended by the manufacturer could cause a free flap. In the present study, all procedures were done strictly according to the manufacturer’s instructions, but FF still occurred. The users’ manual includes guidelines for safe use of the MK but can not completely prevent flap complications. It can be physically understood that very flat rings or very thick ones, as well as a high stop value, have a higher tendency to cause an FF.

From all of the LASIK procedures included in this study, 152 eyes (0.276%) were complicated with an FF. The actual incidence was significantly higher (3.704%) when using nomogram approved combinations of a very flat ring of (-1) and a high stop value of 8.5 mm or another recommended combination of (+2) ring and 8.5 mm stop (2.703%) (Fig 3). These extreme incidences in the first combination are probably caused by trying to treat hyperopic eyes with flat corneas with indicated (-1) ring while aiming at a large ablation area trying to avoid regression and compensating for a nasal angle Kappa using a high stop value of 8.5. Flat corneas are knows to have increased FF risk. The second combination of (+2) ring is indicated for very steep corneas, which in attempting to reach a large ablation zone may sacrifice the hinge. It is physically obvious to understand why a high stop value might be too high for the given ring and cornea and cut through the cap completely. The odd ratio (OR) of a high stop of 8.5 was almost 30 times higher than a short 7.5 mm stop. The effect of the ring height is more difficult to understand. The manufacturer tries to adapt the thicker rings to a stop value and avoid a complete chopping of the steep corneal apex protruding above the ring upper surface. Still, a thicker ring was found to be a risk factor for FF. A (+2) ring is extremly risky with an OR of 3.52 compared to ring (0) and an OR of almost 10 compared to a +1 ring (Table 2).

Other not-so-obvious parameters that could affect the FF incidence are corneal thickness, keratometry and surgeons’ experience with this MK. These possible factors were analyzed in the present study.

The corneal thickness was found to correlate inversly with FF incidence. This may be explained clinically if the assumption is made that thinner flaps also have thinner, less tear-resistant hinges. Different from FSL flaps with their vertical borders, the MK-produced flaps must thin gradually towards their borders which may cause a thin flap hinge to consist of more soft epithelium and less tear-resistant stroma.

It is well known that thicker corneas produce thicker LASIK flaps. In a study of 263 LASIKs using different pivotal Cariazzo MK [8], the flap thickness was significantly correlated to preoperative central corneal thickness (R = 0.271, P< 0.001) but not with keratometries or manifest refraction.

The mean keratometry was not found per se to be a significant FF risk factor (Table 3). This may be explained by choosing the correct ring size for a given cornea and the accuracy of the ring nomogram. It was the ring size that influenced the OR, not the mean keratometry. The same conclusion is brought by Pallikaris [7], stating that corneal keratometric values are irrelevant to keratome complication risk in general or specifically to FF risk. Mitsutoshi et al. [9] found in 0.14% FF incidence that neither flat and steep keratometries nor vertical and horizontal white-to-white corneal diameters were correlated to the incidence of flap complications in general or FF specifically. The last two studies did not use the SBK MK, but nevertheless, their conclusions also correspond to this study’s finding of keratometry not being related to FF incidence.

Another surprising factor is that the experience of the surgeon did not correlate with the FF incidence. One may assume that all surgeons with at least 500 LASIKs have already gained the needed manuality to properly use this automated MK and that the occurance of FF by even extremely experienced surgeons depends on other parameters beyond a surgeon’s experience.

Mitsutoshi [9] also reported 25 (0.67%) flap complications in 3,751 LASIKs done by 10 young surgeons using five different MKs and not the SBK. Three of the flap complications (0.08% of all LASIKs) were FF and all happened only with a single model of the five MKs (LSK one by Moria, France). Out of 2,198 LASIKs done with this specific MK, 17 (0.77%) were complicated and 0.14% (3/2198) were FF. Each of the young surgeons performed 100 LASIks or less prior to participating in this analysis. The overall incidence of complications was independent of the surgeons’ experience, although 10/12 incomplete flaps occurred in the hands of inexperienced surgeons using a manual MK.

### Predictive model for FF incidence

A predictive model for FF incidence using logistic regression was developed. If a cut-off FF incidence similar to the real incidence of 0.276% is chosen, the FF risk can be predicted with 72% sensitivity and 76% specificity.

Given the low incidence of free flaps, the real probability for the patient to have free flaps when the model predicted it is still very low (Positive predictive Value = 0.87%, less than 1%). Although the cost of the false positives is too high and thus the model can’t be used effectively in a clinical setting, it can give an approximate risk for the patient.

If the main concern is avoiding FF based on the cost of the ablation area, one should avoid large stops and extreme rings and be careful with flat keratometries. On the other hand, avoiding FF at all costs will increase the risk of another more difficult to manage complication: hinge ablation and post operative irregular topography.

The fact that less-experienced surgeons did not have more FFs than more-experienced ones and that FF occurred in the experienced hands in the central quartiles of their experience speaks to good training and for a steep learning curve for this well-designed device.

There are several limitations in this study. As is unavoidable when analyzing a rare complication, only a retrospective study could be performed. This study only evaluated one type of commonly used MK. This model did not evaluate the correlation to patients’ cooperation, lid opening and vacuum pressure, which might be clinically relevant.

## Conclusions

To the best of the authors’ knowledge, this is the first large retrospective analysis of more than 50,000 consecutive LASIKs using a modern MK investigating the factors related to FF. This study quantified these factors and used them in a predictive model. Very flat or thick rings, high stop values and low CCT increase the risk of FF in this modern MK. Surgeons’ experience and mean keratometry are not risk factors for FF.

## References

[1] Linke K. *Complications in Corneal Laser Surgery*.; 2016 https://www.springer.com/de/book/978331941494.

[2] Jensen MK, Fiscella RG, Moshirfar M, Mooney B. Third- and fourth-generation fluoroquinolones: Retrospective comparison of endophthalmitis after cataract surgery performed over 10 years. *J Cataract Refract Surg*. 2008;34(9):1460–1467 doi: 10.1016/j.jcrs.2008.05.045 18721704

[3] Moshirfar M, Gardiner JP, Schliesser JA, et al. Laser in situ keratomileusis flap complications using mechanical microkeratome versus femtosecond laser: Retrospective comparison. *J Cataract Refract Surg*. 2010;36(11):1925–1933. doi: 10.1016/j.jcrs.2010.05.027 21029902

[4] Romero-Diaz-de-Leon L, Serna-Ojeda J, Navas A, Graue-Hernández E, Ramirez-Miranda A. Intraoperative flap complications in lasik surgery performed by ophthalmology residents. *J Ophthalmic Vis Res*. 2016;11(3):263. 27621782 10.4103/2008-322X.188393 PMC5000527

[5] Karabela Y, Muftuoglu O, Gulkilik IG, Kocabora MS, Ozsutcu M. Intraoperative and early postoperative flap-related complications of laser in situ keratomileusis using two types of Moria microkeratomes. *Int Ophthalmol*. 2014;34(5):1107–1114. doi: 10.1007/s10792-014-9919-7 24531872

[6] Carrillo C, Chayet AS, Dougherty PJ, et al. Incidence of complications during flap creation in LASIK using the NIDEK MK-2000 microkeratome in 26,600 cases. *J Refract Surg*. 21(5 Suppl):S655–7. http://www.ncbi.nlm.nih.gov/pubmed/16212299. 16212299 10.3928/1081-597X-20050902-20

[7] Walker MB, Wilson SE. Lower intraoperative flap complication rate with the Hansatome microkeratome compared to the Automated Corneal Shaper. *J Refract Surg*. 16(1):79–82. http://www.ncbi.nlm.nih.gov/pubmed/10693623. 10693623 10.3928/1081-597X-20000101-11

[8] Pallikaris IG, Katsanevaki VJ, Panagopoulou SI. Laser in situ keratomileusis intraoperative complications using one type of microkeratome11No author has any type of financial interest that is related to this article, including stock or ownership of a business entity connected to a product described in. *Ophthalmology*. 2002;109(1):57–63. doi: 10.1016/s0161-6420(01)00862-4 11772580

[9] Paschalis EI, Labiris G, Aristeidou AP, Foudoulakis NC, Koukoula SC, Kozobolis VP. Laser in situ keratomileusis flap-thickness predictability with a pendular microkeratome. *J Cataract Refract Surg*. 2011;37(12):2160–2166. doi: 10.1016/j.jcrs.2011.05.044 21996515

